# Executive function, self-regulation skills, behaviors, and socioeconomic status in early childhood

**DOI:** 10.1371/journal.pone.0277013

**Published:** 2022-11-02

**Authors:** Jorge Cuartas, Emily Hanno, Nonie K. Lesaux, Stephanie M. Jones

**Affiliations:** 1 Harvard Graduate School of Education, Harvard University, Cambridge, Massachusetts, United States of America; 2 Center for the Study of Security and Drugs (CESED), Universidad de los Andes, Bogotá, Colombia; VIVE - The Danish Center for Social Science Research, DENMARK

## Abstract

**Background and objectives:**

Prior research has established steep socioeconomic status (SES) disparities in children’s cognitive skills at kindergarten entry. Yet, few studies have had comprehensive, multi-informant data to examine SES-related differences in foundational social and emotional skills and executive function. The objective of the current study is to systematically examine SES-related differences in young children’s executive function (EF), self-regulation skills, and behaviors.

**Methods:**

The current study analyzed data on 2,309 young children from the Early Learning Study at Harvard (ELS@H). Multi-method (direct-assessment and reports) and multi-informant (parents and early education and care educators) information on children’s executive function, self-regulation skills, and internalizing, externalizing, and adaptive behaviors were used. A parametric framework employing Ordinary Least Squares (OLS) estimation was used to quantify the size of the SES-related differences in this set of children’s foundational social-emotional skills.

**Results:**

On average, there were differences of 0.24–0.45 *SD* for EF, 0.22–0.32 *SD* for self-regulation skills, and 0.27–0.54 *SD* for behaviors favoring children from the highest SES quartile of the distribution of SES relative to children from the lowest quartile. The SES-related differences were consistent across direct assessment, parent reports, and educator reports. Some differences were larger for older children relative to their younger counterparts.

**Conclusions:**

Findings indicate a need for comprehensive intervention efforts well before kindergarten entry aimed at closing early disparities in children’s foundational social and emotional skills and executive function.

## Introduction

Early childhood is a sensitive period of development, when processes of myelination and synaptic pruning shape brain and skill development in response to environmental stimuli [1]. Even before kindergarten entry, children from different socioeconomic backgrounds tend to experience vastly different levels of cognitive and social-emotional stimulation [2, 3] and face adversity (e.g., food-insecurity, violence, parental stress) at unequal rates [4, 5]. These differences in everyday experiences are hypothesized to underlie early gaps in cognitive, social, and emotional development that tend to persist and expand over time [6]. This is particularly troubling as early cognitive, social, and emotional skills are strong predictors of later school achievement, physical and mental health, and social mobility [1, 7–9].

As children enter formal schooling, executive function and social and emotional skills are critical for managing the numerous demands of early learning environments [10, 11]. Multiple disciplines conceptualize and operationalize social and emotional skills in different ways, and there is controversy on the best way to measure (e.g., direct assessment, parent reports, educator reports) these skills early in life [12, 13]. In this study, we focus on executive function and several widely studied foundational social and emotional skills, including self-regulation skills and behaviors [11–14]. EF refers to a set of mental processes governed by the brain’s pre-frontal cortex used to produce adaptive, goal-directed behaviors and override more automatic responses [12, 15]. EF includes working memory, response inhibition, attention shifting (i.e., cognitive flexibility), and attention control [12, 16, 17]. Self-regulation skills refer to a broader set of competencies that integrate EF with emotion-oriented skills and knowledge such as effortful control and emotion-regulation to represent more complex regulatory behaviors in context [11, 12, 16]. Finally, social and emotional behaviors include (1) externalizing behaviors, such as hyperactivity, conduct problems, and aggression, (2) internalizing behaviors, such as symptoms of anxiety, depression, or somatization, and (3) adaptive behaviors or skills, such as adaptability, social skills, and daily behaviors that are essential at home, school, and the community [18].

Given the complex and multifaceted nature of executive function and social and emotional skills, evidence on SES disparities in this area is scarce in comparison to the vast body of evidence on SES and cognitive development [2, 3, 19]. In general, parent and educator reports indicate that children from low SES backgrounds tend to have lower levels of self-regulation skills relative to their peers from higher SES backgrounds [20, 21]. Longitudinal studies also suggest that poverty and financial hardship are strong predictors of performance in EF tasks during early childhood [22]. Moreover, recent evidence documents large and growing SES-disparities in internalizing and externalizing behaviors in two cohorts born 30 years apart in the United Kingdom [23]. To the best of our knowledge, this study is the first to simultaneously examine SES-related differences in EF, self-regulation skills, and behaviors using multi-method, multi-informant approaches.

We employ rich multi-method, multi-informant data to quantify the SES gradient in social and emotional skills and behaviors in the two years prior to kindergarten entry. The data contains information about children’s EF (as measured with direct assessment), self-regulation skills (as reported by trained examiners, parents, and early education and care educators), and behaviors (as reported by parents and educators), making it possible to provide a comprehensive description of early disparities in a variety of foundational social and emotional skills.

## Methods

### Sample and procedures

Data were drawn from the first wave of ELS@H, a longitudinal, statewide study of young children’s development and early education and care experiences in Massachusetts. The original ELS@H sample was designed to produce estimates representative of Massachusetts’ population of 3- and- 4- year-old children. Children were recruited through three methods: 1) they were selected from a household survey conducted with over 95,000 households in 168 randomly selected census block groups; 2) they attended early education and care in the same setting as a child recruited via the household survey; or 3) they attended early education and care in a setting randomly selected from administrative records provided by the Massachusetts Department of Early Education and Care [24].

This sampling procedure yieled a sample of 3,222 children ages 3 to 4 years in the study’s first wave in 2017–2018. Data collection included direct assessments of children’s cognitive, social, and emotional skills (administered between February and June, 2018). Assessments were intended to be completed in 45 minutes and all assessors were instructed to stop assessments at the 45-minute mark. Surveys were also conducted with the parents and early education and care educators of participating children (both administered between April and August, 2018). A total of 2,147 parents completed the parent survey, providing information on 2,309 sampled children (some parents completed surveys for multiple children in the same household). Nearly 90% of respondents were female caregivers (with the the vast majority being mothers). A total of 668 educators and caregivers in 465 early education and care providers responded to the survey, providing information on 2,165 children. The Institutional Review Board of Abt Associates and Harvard University approved of all study procedures. Parents and educators gave informed consent and children assented to direct assessments.

The analytic sample for the present study comprises 2,309 children (out of 3,222; 72%) who had information for at least one of the social and emotional outcomes described below and who had complete information on family income and parental education. Children in the analytic sample were, on average, 46.7 months old at the beginning of the study (*range* = 36–60), 49% were female, and 63% were categorized as White, 6% as Black/African American, 12% as Hispanic/Latinx, 6% as Asian, and 13% as other race/ethnicity according to parents’ reports (Table 1).

**Table 1:** Descriptive statistics (*N* = 2,309).

	N	M.	SD	Min.	Max.
Child’s age in months	2307	46.71	6.65	36.00	60.00
*Executive function*					
MEFS–Total score	1779	43.43	14.85	.00	92.00
Pencil Tap–Proportion Correct	1854	.60	.39	.00	1.00
*Self-regulation skills*					
Leiter–soc/cog raw score	2100	2.72	.49	.00	3.00
Leiter–emo/reg raw score	2100	2.87	.33	.00	3.00
BRIEF–dysregulation (parent)	2076	22.59	6.95	3.00	48.00
BRIEF–dysregulation (educator)	1464	21.13	8.02	1.00	48.00
*Behaviors*					
BESS–externalizing (parent)	2086	15.05	4.20	1.00	36.00
BESS–externalizing (educator)	1466	9.20	3.63	5.00	24.00
BESS–internalizing (parent)	2086	13.01	3.04	1.00	29.00
BESS–internalizing (educator)	1466	9.53	2.93	5.00	24.00
BESS–adaptive skills (parent)	2088	27.28	5.24	2.00	36.00
BESS–adaptive skills (educator)	1466	19.58	3.67	6.00	24.00
Total # of people in household	2300	4.30	1.38	.00	32.00
	N	%			
Child’s gender (1 = Female)	2265	48.92			
*Child’s race/ethnicity*	2260				
White		63.05			
Black/African American		5.71			
Hispanic/Latinx		12.39			
Asian		6.24			
Other race/ethnicity		12.61			
*Total household annual income*	2309				
$10k or less		5.89			
$10,0001 to $20,000		6.28			
$20,001 to $30,000		6.76			
$40,001 to $50,000		5.41			
$40,001 to $50,000		4.59			
$50,001 to $75,000		10.00			
$75,001 to $100,000		9.74			
$100,001 to $125,000		10.39			
$125,001 to $150,000		9.87			
$150,001 to $200,000		13.12			
$201,000 to $250,000		7.88			
More than $250,000		10.05			
*Parent education level*	2309				
Less than high school		4.94			
High school		6.70			
Vocational/tech, Some college		14.90			
Associate’s		7.90			
Bachelor’s, some graduate		29.67			
Master’s, Doctoral, Professional Degree		35.89			
Parent lives with spouse/partner	2298	81.42			
*Early education and care provider type*	2309				
Community-based Center Care (CCC)		35.04			
Head Start (HS)		16.67			
Licensed Family Child Care (FCC)		12.17			
Parental Care (PC)		5.46			
Public School Preschool (PSP)		14.51			
Unlicensed Non-Relative Care (UNC)		4.16			
Unlicensed Relative Care (URC)		12.00			

### Measures

#### SES

Information on parental education and family income, two key components of SES [3], were collected in the parent survey. Parents indicated their highest education level from the following categories: 1) less than high school, 2) high school, 3) some college, 4) Associate’s degree, 5) Bachelor’s degree, and 6) graduate degree. Parents reported their family annual income among the following categories: 1) $10k or less, 2) between 10k and 20k, 3) between 20k and 30k, 4) between 30k and 40k, 5) between 40k and 50k, 6) between 50k and 75k, 7) between 75k and 100k, 8) between 100k and 125k, 9) between 125k and 150k, 10) between 150k and 200k, 11) between 200k and 250k, and 12) more than 250k. Drawing on prior studies and procedures applied in the Early Childhood Longitudinal Study [2, 25], principal component analysis (PCA) was used to compute a composite SES index that is the first principal component of parental education and family income. PCA is widely used to estimate SES or wealth indices in household surveys, including the Multiple Indicators Cluster Surveys and the Demographic Health Surveys [26], by extracting the largest amount of variance or information shared by all of the included variables in the first component [27]. The first principal component explained 83.9% of the total variance. The composite SES index was divided into quartiles, where quartile 1 represents the lowest and quartile 4 the highest SES group. Table 2 presents summary statistics for all study variables, including the outcome variables (described below) by SES quartile. There are significant differences in ethnicity composition, total household income, parent education level, and provider care setting type between the different SES quartiles.

**Table 2:** Descriptive statistics by SES quartile (N = 2309).

	Q1		Q2		Q3		Q4	
	Mean/%	SD	Mean/%	SD	Mean/%	SD	Mean/%	SD
Child’s age in months	47.69	6.59	47.15	6.68	46.37	6.76	45.46	6.35
*Executive function*	55.69	6.65	54.41	6.94	53.66	6.84	52.72	6.61
MEFS—Total score	57.89	6.76	56.65	6.79	55.72	6.88	54.82	6.48
Pencil Tap—Proportion Correct	57.46	6.60	56.71	6.63	55.90	6.80	54.30	6.42
*Self-regulation skills*	38.83	14.24	41.42	15.21	46.37	14.88	46.43	13.58
Leiter—soc/cog raw score	.50	.41	.59	.40	.67	.36	.64	.38
Leiter—emo/reg raw score	15.81	5.45	14.80	3.93	14.92	3.56	14.64	3.38
BRIEF–dysregulation (parent)	9.78	4.02	9.13	3.64	8.99	3.44	8.92	3.36
BRIEF–dysregulation (provider)	13.02	3.63	12.85	2.96	13.18	2.75	12.99	2.71
*Behaviors*	9.85	2.98	9.57	2.99	9.30	2.91	9.42	2.82
BESS–externalizing (parent)	22.84	8.18	22.76	6.97	22.55	6.38	22.17	6.01
BESS–externalizing (provider)	22.64	9.00	21.44	8.02	20.19	7.48	20.33	7.32
BESS–internalizing (parent)	25.24	6.29	27.44	5.24	28.00	4.42	28.51	4.03
BESS–internalizing (provider)	18.45	3.88	19.24	3.74	20.11	3.47	20.45	3.23
BESS–adaptive skills (parent)	2.62	.60	2.69	.51	2.75	.47	2.84	.33
BESS–adaptive skills (provider)	2.82	.43	2.87	.34	2.89	.30	2.92	.21
Total # of people in household	4.37	1.91	4.38	1.40	4.20	1.00	4.26	.88
Child’s gender (1 = Female)	49.57		46.25		51.22		48.65	
*Ethnicity*								
White	32.59		62.33		80.70		78.57	
Black/African American	11.32		7.36		2.96		0.58	
Hispanic/Latinx	33.28		12.50		1.91		0.39	
Asian	6.69		4.97		5.39		8.11	
Other race/ethnicity	16.12		12.84		9.04		12.36	
*Total household annual income*								
10k or less	22.17		0.17					
10,0001 to 20,000	20.36		3.55					
20,001 to 30,000	20.69		5.08					
40,001 to 50,000	10.84		9.98					
40,001 to 50,000	9.69		7.95					
50,001 to 75,000	12.81		18.27		7.73			
75,001 to 100,000	2.63		23.52		12.03			
100,001 to 125,000	.82		20.64		19.42			
125,001 to 150,000			4.57		34.54			
150,001 to 200,000			4.06		14.95		36.43	
201,000 to 250,000			1.02		10.65		21.63	
More than 250,000			1.18		0.69		41.94	
*Parent education level*								
Less than high school	18.72							
HS	32.84		0.85					
Vocational/tech, Some college	39.90		17.09					
Associate’s	6.57		21.32		1.37			
Bachelor’s, some graduate	1.97		56.35		40.89		14.99	
Master’s, Doctoral, Professional Degree			4.40		57.73		85.01	
Parent lives with spouse/partner	57.64		81.36		91.88		97.15	
*Provider care setting type*								
Community-based Center Care (CCC)	17.08		27.41		43.81		54.65	
Head Start (HS)	47.62		14.55		1.37		0.19	
Licensed Family Child Care (FCC)	10.02		12.18		15.46		11.01	
Parental Care (PC)	6.08		9.14		4.47		1.71	
Public School Preschool (PSP)	11.33		19.63		17.01		9.68	
Unlicensed Non-Relative Care (UNC)	.49		2.20		3.61		11.20	
Unlicensed Relative Care (URC)	7.39		14.89		14.26		11.57	

#### Executive function

Two instruments were administered to assess EF. First, The Minnesota Executive Function Scale (MEFS) [28], which is designed to capture working memory, inhibitory control, and set shifting using graphics, avatars, and child-directed instructions in an iPad/tablet app. The MEFS is adaptive to children’s skills, with a starting point determined by children’s age, and takes between 2 to 7 minutes to complete. Scores from the MEFS are computed using an algorithm that considers accuracy and response time with higher scores representing greater EF. Second, The Pencil Tap (PT) task [29] measured the inhibitory control component of EF. In the task, assessors tapped a pencil once or twice and the child was required to inhibit their dominant response to tap the opposite number of times across a total of 16 trials. Scores indicated the proportion of correct trials and exhibited adequate reliability in the sample (Cronbach’s alpha [α] = 0.88).

#### Self-regulation skills

Two instruments were used to capture children’s self-regulation skills. First, assessors used the Leiter-3 Examiner Rating Scale [30] to score the frequency of 56 different statements about children’s skills observed during the assessments, using a scale from 0 for “rarely/never” to 3 for “usually/always.” This study uses the cognitive/social (related to children’s attention and impulsivity) and emotion/regulation (related to emotional regulation and anxiety) composite scores. Scores were generated by averaging item-level scores such that higher scores indicated greater self-regulation skills. The cognitive/social (*α* = 0.98) and emotion/regulation (*α* = 0.97) composite scores exhibited adequate psychometric properties in the sample.

Second, parents and educators reported on their children’s skills using the Behavior Rating Inventory of Executive Function (BRIEF) screening version [31] The BRIEF asks reporters to note the frequency of 12 child behaviors that inicate dysregulation (e.g., having trouble putting the brakes on his/her actions; not realizing that certain actions bother other; reacting more strongly to situation than other children) in the prior three months on a scale from 1 for “never occurs” to 4 “almost always occurs.” A total of 2,076 and 1,464 children had complete information on the BRIEF as reported by parents and educators, respectively. (A higher score represented more dysregulation, i.e., lower self-regulation skills).

#### Behaviors

The Behavioral and Emotional Screening System (BESS) was used in the parent and educator surveys to measure children’s behavioral strengths and difficulties [32]. Parents and educators reported the frequency of various child behaviors in the prior three months on a scale from one for “never occurs” to four “almost always occurs.” Prior psychometric assessment of the BESS [18, 33] were used to define three subscales: externalizing risk, internalizing risk, and adaptive behaviors. Scores were generated by averaging item-level scores such that higher scores indicated greater externalizing, internalizing, and adaptive behaviors on each of the three subscales. The externalizing risk subscale comprised 9 items in the parent form (*α* = .85) and 6 items in the educator (*α* = 0.91) form related to externalizing behaviors, such as hyperactivity, aggression, and conduct problems. The internalizing risk subscale comprises 8 items in the parent form (*α* = .74) and 6 items in the educator form (*α* = .83) that represent internalizing problems, such as anxiety and depression. Finally, the adaptive behaviors subscale comprised 9 items in the parent form (*α* = .85) and 6 items in the educator (*α* = .85) that assessed children’s adaptability, social skills, and other behaviors important for daily functioning.

#### Covariates

Information about children’s age, gender, race/ethnicity, number of household members, and presence of the respondent’s spouse was gathered using the parent survey. Finally, information about the type of provider each child attended was captured from the parent survey and confirmed using a variety of sources, and operationalized as the following categories of provider types: community-based center care, Head Start, licensed family child care, parental care, public school prekindergarten, unlicensed non-relative care, and unlicensed relative care.

### Analysis

All child outcomes were transformed into z-scores with respect to the sample mean and standard deviation for comparability of SES quartiles across all social and emotional constructs. Consequently, all estimates can be interpreted in standard deviations within the sample. A parametric framework employing Ordinary Least Squares (OLS) estimation, which relies on the assumptions of linear regression, was used to quantify the size of the SES-related differences in children’s social-emotional skills. Specifically, the z-score of child *i* on social-emotional outcome *j* was estimated using the following specification:

$$zY_{i}^{s}=\alpha +\sum_{q=2}^{4}\beta_{q}^{j}Q_{qi}+\gamma_{1i}^{j}age_{i}+\gamma_{2i}^{j}{age}_{i}^{2}+\gamma_{3i}^{j}female_{i}+\gamma_{4i}^{j}{spouse}_{i}+\gamma_{5i}^{j}{members}_{i}+\sum_{q=2}^{5}\theta_{q}^{j}race+\sum_{q=2}^{7}\kappa_{q}^{j}providertype+\epsilon_{i}^{j}$$

In this model, *Q*_*qi*_ was the *qth* quartile of the SES distribution. Moreover, *age*_*i*_ was the age in months of child *i*, ${age}_{i}^{2}$ was age in months squared to model marginally decreasing developmental plasticity as children get older [1, 6, 9], *female*_*i*_ was an indicator of the gender of child *i*. In addition, *spouse*_*i*_ was an indicator that equals one if the spouse of the parent who responded the survey lived at the house, *members*_*i*_ was the total number of household members, and *race* was a vector of binary variables indicating child *i*’s parent-reported race. In the model, *providertype* was a vector of binary variables indicating child *i*’s provider type, which was included to reduce potential confounding due to differences in early education and care provider type. $\beta_{q}^{j}$ was the vector of coefficients of interest, which represented the difference in the average z-score in the social-emotional outcome *j* of children in quartile *q* relative to the average z-score for children in the first SES quartile. $\epsilon_{i}^{j}$ represented residual variance and includes unobservable child and household characteristics.

Second, differences by child age on SES differences were examined. Fractional-polynomial plots, which allow for flexible parameterization, were used to visually inspect differences in outcomes between children in families in Q1 and Q4 by age. Subsequently, the same parametric framework was used to test whether SES-related differences significantly varied by age-group (children aged 36–42 months, 43–50, and 51–60). All analyses were conducted in Stata/MP 16.1 [34].

## Results

**Fig 1:**
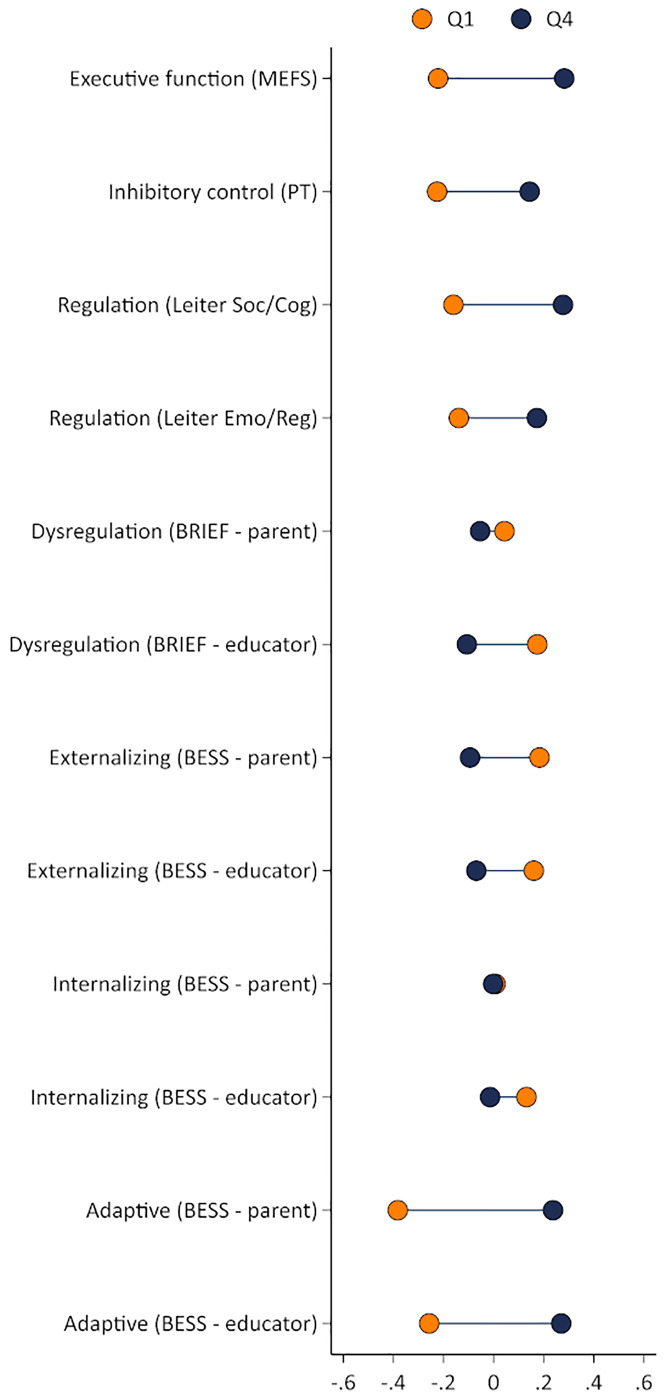
Average standardized score for SES quartiles 1 and 4.

Fig 1 provides a descriptive illustration showing overall unadjusted SES-related differences in children’s executive function and social and emotional outcomes favoring children in the highest quartile of the SES distribution (see S1 Table for formal mean difference tests). In particular, statistically significant differences were observed across the majority of measures. Notable exceptions were self-regulation skills on the BRIEF and internalizing risk, as reported by parents, although differences were observed on these constructs as reported by educators.

Tables 2–4 summarize results for the parametric framework used to quantify the size of the average SES-related differences on children’s EF, self-regulation skills, and behaviors. Average SES-related differences were identified for executive function (Table 3), as measured by the MEFS (0.43 SD for Q3 and 0.45 SD for Q4 relative to Q1) and the Pencil Tap (0.28 SD for Q3 and 0.24 SD for Q4 relative to Q1). Similarly, SES-related differences were identified for self-regulation skills (Table 4), specifically for the Leiter cognitive/social (0.16 for Q3 and 0.32 for Q4 relative to Q1) and emotion/regulation (0.22 for Q4 relative to Q1) subscales, and for dysregulation according to parent (-0.20 SD for Q4 relative to Q1) and educator reports (-0.29 for Q3 and -0.32 for Q4 relative to Q1). Finally, there were sizable SES-related differences in children’s behaviors (Table 5), specifically in externalizing risk as reported by parents (-0.26 SD for Q2, -0.24 SD for Q3, and -0.32 SD for Q4 relative to Q1) and educators (-0.18 SD for Q2, -0.22 SD for Q3, and -0.27 SD for Q4 relative to Q1) and adaptive behaviors as reported by parents (0.40 SD for Q2, 0.46 SD for Q3, and 0.53 SD for Q4 relative to Q1) and educators (0.16 SD for Q2, 0.30 SD for Q3, and 0.43 SD for Q4 relative to Q1). No differences were identified in internalizing risk between SES quartiles according to parent and educator reports. Sensitivity checks reveal that these findings are largely robust when restricting the sample to children who have complete information for all outcomes (S2–S4 Tables).

**Table 3:** Average SES effects in executive function and inhibitory control.

	(1)	(2)
	EF (MEFS)	Inhibitory control (PT)
Q2	0.13	0.12
	(-0.01–0.26)	(-0.00–0.25)
Q3	0.43***	0.28***
	(0.28–0.57)	(0.14–0.41)
Q4	0.45***	0.24**
	(0.29–0.60)	(0.10–0.39)
N	1,739	1,811
R-sq.	0.24	0.24

Note. 95% confidence intervals in parentheses. All models include as covariates age, age-sq, gender, race/ethnicity, respondent’s spouse lives at home, total household members, provider type

*** *p* < .001,

** *p* < .01,

* *p* < .05

**Table 4:** Average SES effects in self-regulation skills.

	(1)	(2)	(3)	(4)
	Regulation (Leiter-Cog/Soc)	Regulation (Leiter Emo/Reg)	Dysregulation (BRIEF–parent)	Dysregulation (BRIEF–educator)
Q2	0.09	0.10	-0.09	-0.16*
	(-0.03–0.22)	(-0.03–0.23)	(-0.22–0.04)	(-0.32–-0.00)
Q3	0.16*	0.12	-0.14	-0.29***
	(0.02–0.29)	(-0.02–0.26)	(-0.28–0.01)	(-0.47–-0.12)
Q4	0.32***	0.22**	-0.20*	-0.32***
	(0.18–0.47)	(0.07–0.37)	(-0.35–-0.04)	(-0.50–-0.14)
N	2,046	2,046	2,066	1,425
R-sq.	0.10	0.05	0.06	0.09

Note. 95% confidence intervals in parentheses. All models include as covariates age, age-sq, gender, race/ethnicity, respondent’s spouse lives at home, total household members, provider type

*** *p* < .001,

** *p* < .01,

* *p* < .05

**Table 5:** Average SES effects in behaviors.

	(1)	(2)	(3)	(4)	(5)	(6)
	Externalizing (BESS–parent)	Externalizing (BESS—educator)	Internalizing (BESS–parent)	Internalizing (BESS–educator)	Adaptive (BESS–parent)	Adaptive (BESS–educator)
Q2	-0.26***	-0.18*	-0.09	-0.10	0.40***	0.16*
	(-0.39–-0.13)	(-0.34–-0.02)	(-0.22–0.04)	(-0.27–0.06)	(0.27–0.53)	(0.00–0.31)
Q3	-0.24**	-0.22*	-0.01	-0.16	0.46***	0.30***
	(-0.38–-0.09)	(-0.40–-0.05)	(-0.16–0.14)	(-0.34–0.03)	(0.31–0.60)	(0.13–0.47)
Q4	-0.32***	-0.27**	-0.08	-0.07	0.53***	0.42***
	(-0.47–-0.17)	(-0.46–-0.08)	(-0.23–0.08)	(-0.27–0.12)	(0.38–0.68)	(0.24–0.60)
N	2,075	1,427	2,075	1,427	2,077	1,427
R-sq.	0.05	0.08	0.01	0.03	0.08	0.12

Note. 95% confidence intervals in parentheses. All models include as covariates age, age-sq, gender, race/ethnicity, respondent’s spouse lives at home, total household members, provider type

*** *p* < .001,

** *p* < .01,

* *p* < .05

**Fig 2:**
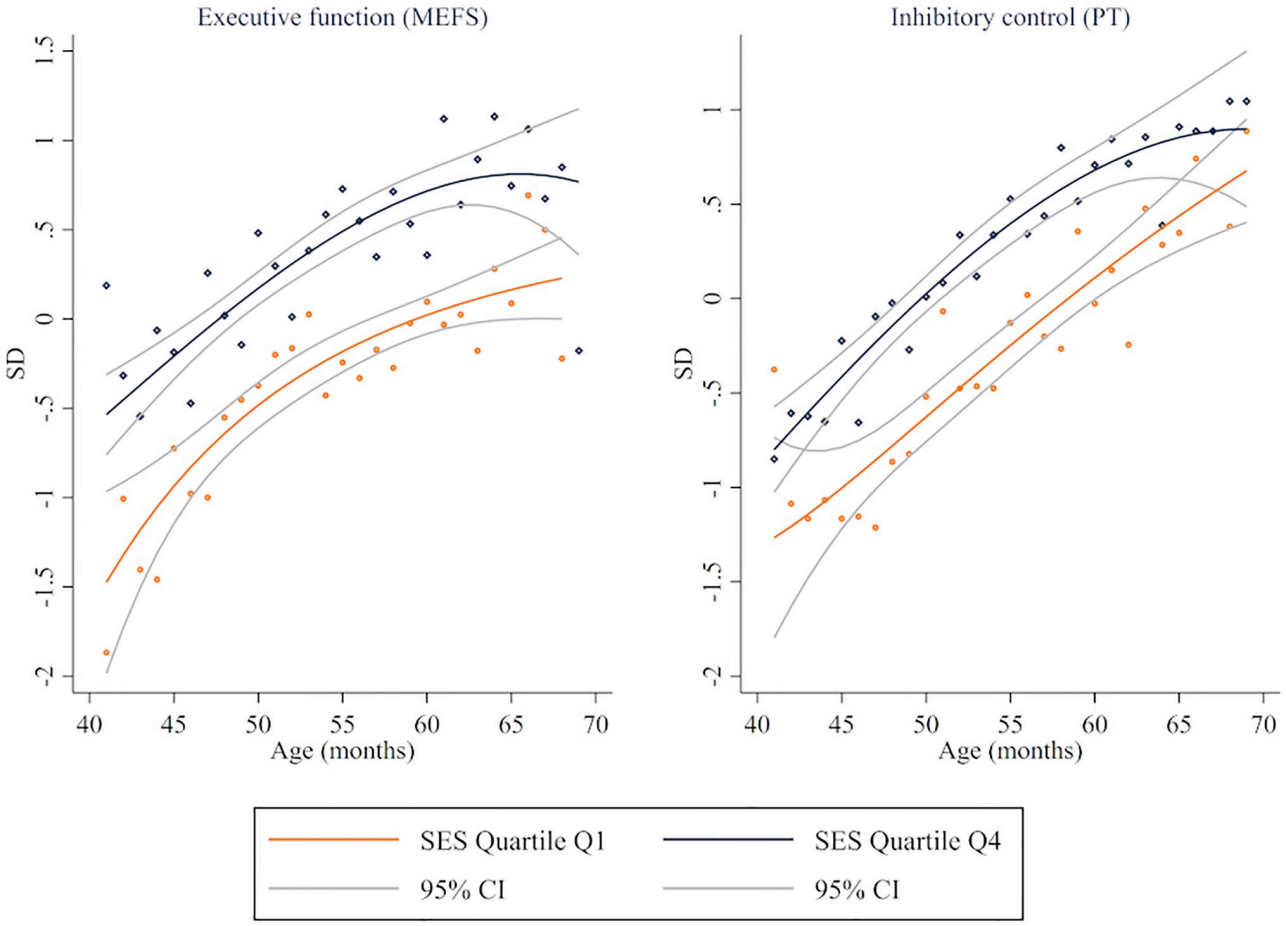
Executive function by SES quartile. Fractional-polynomial plots by developmental domain.

**Fig 3:**
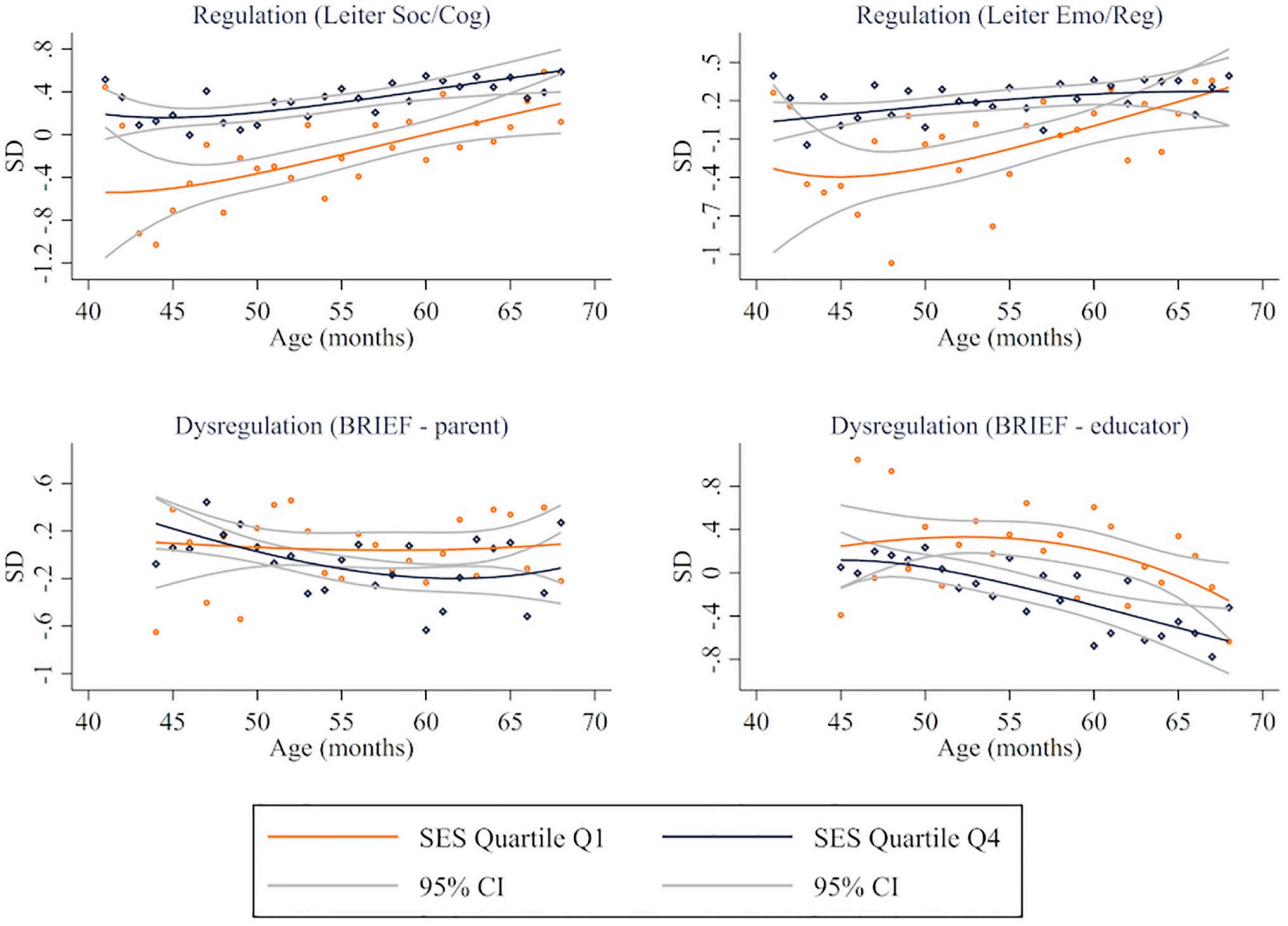
Regulatory-related skills by age by SES quartile. Fractional-polynomial plots by developmental domain.

**Fig 4:**
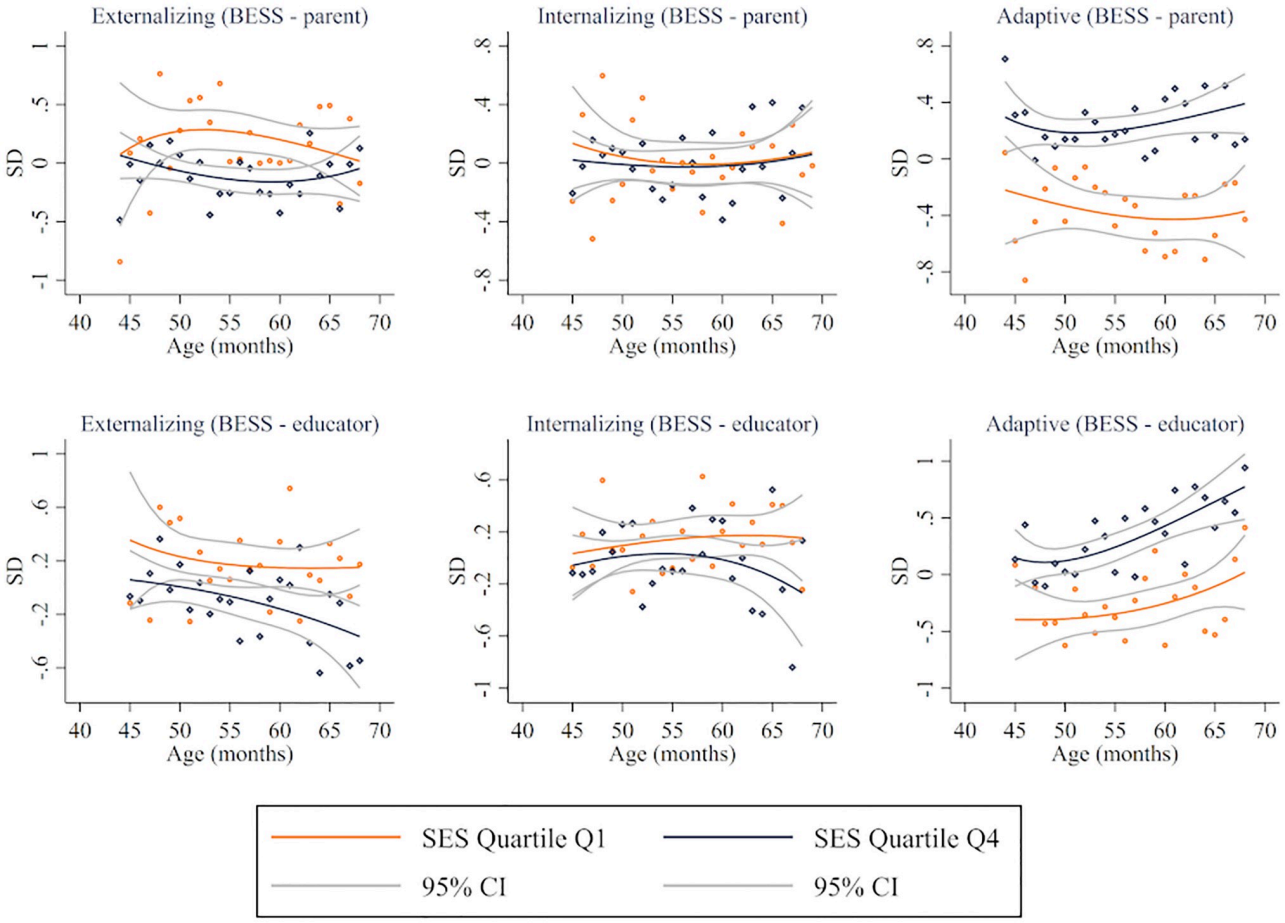
Behaviors by age by SES quartile. Fractional-polynomial plots by developmental domain.

Regarding differences by age, Figs 2–4 illustrate fractional-polynomial regressions showing differences in outcomes between children in families in Q1 and Q4 by age along with 95 percent confidence intervals. The figures show relatively consistent differences for children of different ages, with more uncertainty for the youngest and oldest children due to fewer observations. Consistent with the results presented above, sizable differences were identified in EF, self-regulation skills (except in parents’ reports), and behaviors (except in internalizing risk). However, a parametric approach (see S5–S13 Tables) indicates that the difference between Q4 and Q1 increases, on average, with age for EF as measured by the MEFS (0.30 SD for children aged 36–42 and 0.46 SD for 51–60), dysregulation as reported by educators (-0.28 SD for 36–42 and -0.57 SD for 51–60), externalizing risk as reported by educators (-0.27 SD for 36–42 and -0.43 for 51–60), internalizing risk as reported by educators (-0.14 SD for 36–42 and -0.42 SD for 51–60), and adaptive skills as reported by parents (0.51 SD for 36–42 and 0.71 for 51–60) and educators (0.19 SD for 36–42 and 0.61 SD for 51–60).

## Discussion

This study documented sizable SES-related differences in executive function and foundation social and emotional skills, as measured using direct assessments, parent reports, and educator reports, in young children participating in a statewide study in Massachusetts. Consistent with this set of findings, extensive research shows children from different SES backgrounds tend to experience dissimilar risk and protective factors prenatally and in their first years of life [2–6]. For example, low-SES families often face financial constraints and lack access to community resources and high-quality early education and care, limiting children’s opportunities for critical cognitive, social, and emotional stimulation and supports that higher-income peers tend to experience [3, 6, 35, 36]. Many families from low-SES backgrounds are also exposed to additional adversities and chronic stressors such as food-insecurity, poor health and nutrition, income-insecurity, parental depression, and community violence, among others, at higher rates that high-SES families [3, 21, 35, 37–39].

The significant disparities in everyday experiences between children from different SES backgrounds are likely at play in the observed differences in children’s executive function and foundational social and emotional skills. Neural circuits underlying executive function and self-regulation skills in particular have a protacted course of development [40]. Despite the prolonged window of malleability of EF and self-regulation skills, several fields, including pediatrics, developmental cognitive neuroscience, and developmental psychology, have demonstrated that neural circuits underlying these skills are particularly responsive and sensitive to experiences and environments (both positive and negative) during early childhood [1, 41]. Electroencephalogram (EEG) and functional magnetic resonance imaging studies (fMRI) have shown a shift in activation from the whole brain to frontal regions [41] and increased patterns of activation of the prefrontal cortex (PFC; including the frontal sulcus) from infancy to ages 4–5 years in EF tasks [42], likely due to myelination that occurs at a fast pace during these years to increase the efficiency and integration of information. Consistent with this neurodevelopmental perspective, neuroimaging studies indicate that differences in PFC activation in EF tasks between low-SES and high-SES are evident even before kindergarten entry [43].

Early disparities in executive function and social and emotional skill development can persist and widen across the lifespan, having long-lasting consequences for children’s academic and social trajectories. During the preschool period children build several EF and self-regulation skills such as the ability to represent problems and plan solutions, execute problem-solving actions, assess the outcomes of their plans, and use such assessments as feedback for future behavior [41]. Consequently, the unequal acquisition of these skills along the SES spectrum can place low-SES children at a substantial early disadvantage, making it more difficult for them to maintain positive relationships with peers and educators in academic contexts, employ EF and self-regulation skills to engage in and perform well in academic tasks, and, eventually, to acquire other more complex cognitive and social-emotional skills [22, 39, 41]. In the long-term, early disparities in social and emotional development could translate into differences in educational achievement, employment, economic welfare, and mental health [6, 7, 44–46].

A major strength of the current study was the use of multi-method, multi-informant data on EF, self-reguation skills, and behaviors, which allow us to comprehensively characterize children’s early social and emotional development. Nonetheless, the study has limitations that inform future directions for research. As in prior research on SES-related disparities in children’s development [3], this study cannot rule out the influence of potential confounders in the association between SES and children’s development, including the home learning environment, parent-child interactions, child and parent stress, and/or access to social services. Future studies must leverage experimental and longitudinal designs to better understand the causal influence of SES on social and emotional development across the life course. Second, this study does not examine the mechanisms or mediators underlying the links between SES and development. Future research should explore the role of neurophysiological factors and other potential mediators to better understand the mechanisms through which SES might impact children’s social and emotional development. Finally, while the direction of results is consistent when considering parent and provider ratings, future psychometric work should explore potential systematic biases in the reports of parents and teachers (e.g., examine to whether and what degree parents and/or teachers over- or under-estimate children’s skills).

In sum, this study, along with prior evidence about significant SES disparities in children’s early experiences and cognitive development [2, 3, 47], suggest the need for comprehensive supports for children from low-SES backgrounds. Large-scale integrated parenting programs that provide supports related to health, nutrition, stimulation, and positive discipline can reduce multiple risk factors, benefit child and family outcomes, and have the potential to be cost-effective [48–50]. Indeed, parenting programs have been successful in supporting children’s EF, self-regulation skills, and in reducing externalizing behaviors in at-risk children, with effect sizes of one-quarter to half of a standard deviation [51], which would be enough to close some of the gaps between children from low and high SES backgrounds identified in the present study. Other parenting programs that integrated psychosocial support and nutrition interventions have had similar sizable effects [48, 52]. Pediatricians can also play a significant role by increasing parents’ awareness of the importance of social and emotional development, suggesting easy-to-do activities and games to promote the development of EF and self-regulation at home [53], and referring families to specialized supports and services when needed. Doing so is particularly relevant in the time of COVID-19, when the exacerbaton of SES-related risk factors [54, 55], can impose additional threats to the healthy development and well-being of children.

## Supporting information

S1 Table Mean difference in average standardized scores for SES quartiles 1 and 4. (DOCX)

S2 Table Average SES effects in executive function using sample without missing data. (DOCX)

S3 Table Average SES effects in self-regulation skills using sample without missing data. (DOCX)

S4 Table Average SES effects in behaviors using sample without missing data. (DOCX)

S5 Table Average SES effects in executive function for children aged 36–42 months. (DOCX)

S6 Table Average SES effects in self-regulation skills for children aged 36–42 months. (DOCX)

S7 Table Average SES effects in behaviors for children aged 36–42 months. (DOCX)

S8 Table Average SES effects in executive function for children aged 43–50 months. (DOCX)

S9 Table Average SES effects in self-regulation skills for children aged 43–50 months. (DOCX)

S10 Table Average SES effects in behaviors for children aged 43–50 months. (DOCX)

S11 Table Average SES effects in executive function for children aged 51–60 months. (DOCX)

S12 Table Average SES effects in self-regulation skills for children aged 51–60 months. (DOCX)

S13 Table Average SES effects in behaviors for children aged 51–60 months. (DOCX)
