# Supplementary material for: Executive function, self-regulation skills, behaviors, and socioeconomic status in early childhood
Source: PLoS One. 2022 Nov 2;17(11):e0277013. doi: 10.1371/journal.pone.0277013 (PMC9629624; doi:10.1371/journal.pone.0277013)
Supplement: S1 Table — (DOCX) [file pone.0277013.s001.docx]

S1 Table. Mean difference in average standardized scores for SES quartiles 1 and 4.

|  | Mean | | Difference |
| --- | --- | --- | --- |
|  | Q1 | Q4 | Q4-Q1 |
| Executive function (MEFS) | -0.22  (0.05) | 0.28  (0.04 | 0.50*** |
| Inhibitory control (PT) | -0.23  (0.05) | 0.14  (0.04) | 0.37*** |
| Regulation (Leiter Cog/Soc) | -0.16  (0.05) | 0.28  (0.03) | 0.44*** |
| Regulation (Leiter Emo/Reg) | -0.14  (0.06) | 0.17  (0.03) | 0.31*** |
| Dysregulation (BRIEF - parent) | 0.04  (0.05) | -0.05  (0.04) | -0.09 |
| Dysregulation (BRIEF - provider) | 0.17  (0.06) | -0.11  (0.05) | -0.28*** |
| Externalizing (BESS - parent) | 0.18  (0.06) | -0.09  (0.04) | -0.28*** |
| Externalizing (BESS - provider) | 0.16  (0.06) | -0.07  (0.05) | -0.23** |
| Internalizing (BESS - parent) | 0.01  (0.05) | -0.00  (0.04) | -0.01 |
| Internalizing (BESS - provider) | 0.13  (0.05) | -0.01  (0.05) | -0.15* |
| Adaptive (BESS - parent) | -0.38  (0.05) | 0.24  (0.04) | 0.62*** |
| Adaptive (BESS - provider) | -0.26  (0.05) | 0.27  (0.05) | 0.53*** |

Note. Standard errors in parentheses.

*** p<0.001, ** p<0.01, * p<0.05
