# Supplementary material for: Executive function, self-regulation skills, behaviors, and socioeconomic status in early childhood
Source: PLoS One. 2022 Nov 2;17(11):e0277013. doi: 10.1371/journal.pone.0277013 (PMC9629624; doi:10.1371/journal.pone.0277013)
Supplement: S2 Table — (DOCX) [file pone.0277013.s002.docx]

S2 Table. Average SES effects in executive function using sample without missing data

|  | (1) | (2) |
| --- | --- | --- |
| VARIABLES | EF (MEFS) | Inhibitory control (PT) |
|  |  |  |
| Q2 | 0.21* | 0.25** |
|  | (0.03 - 0.39) | (0.07 - 0.43) |
| Q3 | 0.46*** | 0.47*** |
|  | (0.27 - 0.66) | (0.27 - 0.66) |
| Q4 | 0.52*** | 0.42*** |
|  | (0.31 - 0.72) | (0.21 - 0.62) |
|  |  |  |
| N | 955 | 955 |
| R-sq. | 0.21 | 0.25 |

Note. 95% confidence intervals in parentheses. All models include as covariates age, age-sq, gender, race/ethnicity, respondent’s spouse lives at home, total household members, provider type

*** *p*<.001, ** *p*<.01, * *p*<.05
