# Supplementary material for: Executive function, self-regulation skills, behaviors, and socioeconomic status in early childhood
Source: PLoS One. 2022 Nov 2;17(11):e0277013. doi: 10.1371/journal.pone.0277013 (PMC9629624; doi:10.1371/journal.pone.0277013)
Supplement: S3 Table — (DOCX) [file pone.0277013.s003.docx]

S3 Table. Average SES effects in self-regulation skills using sample without missing data

|  | (1) | (2) | (3) | (4) |
| --- | --- | --- | --- | --- |
| VARIABLES | Regulation (Leiter-Cog/Soc) | Regulation (Leiter Emo/Reg) | Dysregulation (BRIEF - parent) | Dysregulation (BRIEF - provider) |
|  |  |  |  |  |
| Q2 | 0.06 | 0.04 | -0.01 | -0.16 |
|  | (-0.10 - 0.22) | (-0.12 - 0.20) | (-0.21 - 0.20) | (-0.35 - 0.04) |
| Q3 | 0.11 | 0.04 | -0.05 | -0.24* |
|  | (-0.06 - 0.28) | (-0.13 - 0.21) | (-0.27 - 0.17) | (-0.45 - -0.04) |
| Q4 | 0.23* | 0.11 | -0.16 | -0.25* |
|  | (0.04 - 0.41) | (-0.07 - 0.29) | (-0.39 - 0.08) | (-0.47 - -0.03) |
|  |  |  |  |  |
| N | 955 | 955 | 955 | 955 |
| R-sq. | 0.09 | 0.04 | 0.07 | 0.10 |

Note. 95% confidence intervals in parentheses. All models include as covariates age, age-sq, gender, race/ethnicity, respondent’s spouse lives at home, total household members, provider type

*** *p*<.001, ** *p*<.01, * *p*<.05
