# Supplementary material for: Executive function, self-regulation skills, behaviors, and socioeconomic status in early childhood
Source: PLoS One. 2022 Nov 2;17(11):e0277013. doi: 10.1371/journal.pone.0277013 (PMC9629624; doi:10.1371/journal.pone.0277013)
Supplement: S4 Table — (DOCX) [file pone.0277013.s004.docx]

S4 Table. Average SES effects in behaviors using sample without missing data

|  | (1) | (2) | (3) | (4) | (5) | (6) |
| --- | --- | --- | --- | --- | --- | --- |
| VARIABLES | Externalizing (BESS - parent) | Externalizing (BESS -provider) | Internalizing (BESS - parent) | Internalizing (BESS - provider) | Adaptive (BESS - parent) | Adaptive (BESS - provider) |
|  |  |  |  |  |  |  |
| Q2 | -0.33** | -0.14 | -0.08 | -0.09 | 0.31** | 0.09 |
|  | (-0.54 - -0.12) | (-0.34 - 0.05) | (-0.29 - 0.13) | (-0.31 - 0.12) | (0.11 - 0.50) | (-0.09 - 0.28) |
| Q3 | -0.32** | -0.15 | -0.02 | -0.11 | 0.39*** | 0.23* |
|  | (-0.54 - -0.10) | (-0.35 - 0.06) | (-0.25 - 0.20) | (-0.34 - 0.11) | (0.18 - 0.60) | (0.03 - 0.43) |
| Q4 | -0.48*** | -0.19 | -0.19 | -0.06 | 0.54*** | 0.31** |
|  | (-0.72 - -0.25) | (-0.41 - 0.03) | (-0.43 - 0.05) | (-0.29 - 0.18) | (0.32 - 0.76) | (0.10 - 0.52) |
|  |  |  |  |  |  |  |
| N | 955 | 955 | 955 | 955 | 955 | 955 |
| R-sq. | 0.06 | 0.08 | 0.02 | 0.04 | 0.07 | 0.13 |

Note. 95% confidence intervals in parentheses. All models include as covariates age, age-sq, gender, race/ethnicity, respondent’s spouse lives at home, total household members, provider type

*** *p*<.001, ** *p*<.01, * *p*<.05
