# Supplementary material for: Executive function, self-regulation skills, behaviors, and socioeconomic status in early childhood
Source: PLoS One. 2022 Nov 2;17(11):e0277013. doi: 10.1371/journal.pone.0277013 (PMC9629624; doi:10.1371/journal.pone.0277013)
Supplement: S5 Table — (DOCX) [file pone.0277013.s005.docx]

S5 Table. Average SES effects in executive function for children aged 36-42 months

|  | (1) | (2) |
| --- | --- | --- |
| VARIABLES | EF (MEFS) | Inhibitory control (PT) |
|  |  |  |
| Q2 | 0.02 | 0.21 |
|  | (-0.24 - 0.28) | (-0.07 - 0.50) |
| Q3 | 0.44** | 0.50*** |
|  | (0.16 - 0.71) | (0.21 - 0.80) |
| Q4 | 0.30* | 0.38* |
|  | (0.02 - 0.59) | (0.07 - 0.68) |
|  |  |  |
| N | 474 | 496 |
| R-sq. | 0.19 | 0.14 |

Note. 95% confidence intervals in parentheses. All models include as covariates age, age-sq, gender, race/ethnicity, respondent’s spouse lives at home, total household members, provider type

*** *p*<.001, ** *p*<.01, * *p*<.05
