# Supplementary material for: Executive function, self-regulation skills, behaviors, and socioeconomic status in early childhood
Source: PLoS One. 2022 Nov 2;17(11):e0277013. doi: 10.1371/journal.pone.0277013 (PMC9629624; doi:10.1371/journal.pone.0277013)
Supplement: S6 Table — (DOCX) [file pone.0277013.s006.docx]

S6 Table. Average SES effects in self-regulation skills for children aged 36-42 months

|  | (1) | (2) | (3) | (4) |
| --- | --- | --- | --- | --- |
| VARIABLES | Regulation (Leiter-Cog/Soc) | Regulation (Leiter Emo/Reg) | Dysregulation (BRIEF - parent) | Dysregulation (BRIEF - provider) |
|  |  |  |  |  |
| Q2 | 0.03 | 0.13 | -0.06 | -0.11 |
|  | (-0.25 - 0.31) | (-0.17 - 0.43) | (-0.31 - 0.20) | (-0.43 - 0.20) |
| Q3 | 0.19 | 0.26 | -0.24 | -0.43* |
|  | (-0.11 - 0.49) | (-0.06 - 0.58) | (-0.51 - 0.04) | (-0.76 - -0.09) |
| Q4 | 0.30 | 0.30 | -0.14 | -0.28 |
|  | (-0.02 - 0.61) | (-0.03 - 0.63) | (-0.43 - 0.14) | (-0.63 - 0.06) |
|  |  |  |  |  |
| N | 559 | 559 | 572 | 411 |
| R-sq. | 0.14 | 0.09 | 0.08 | 0.15 |

Note. 95% confidence intervals in parentheses. All models include as covariates age, age-sq, gender, race/ethnicity, respondent’s spouse lives at home, total household members, provider type

*** *p*<.001, ** *p*<.01, * *p*<.05
