# Supplementary material for: Executive function, self-regulation skills, behaviors, and socioeconomic status in early childhood
Source: PLoS One. 2022 Nov 2;17(11):e0277013. doi: 10.1371/journal.pone.0277013 (PMC9629624; doi:10.1371/journal.pone.0277013)
Supplement: S7 Table — (DOCX) [file pone.0277013.s007.docx]

S7 Table. Average SES effects in behaviors for children aged 36-42 months

|  | (1) | (2) | (3) | (4) | (5) | (6) |
| --- | --- | --- | --- | --- | --- | --- |
| VARIABLES | Externalizing (BESS - parent) | Externalizing (BESS -provider) | Internalizing (BESS - parent) | Internalizing (BESS - provider) | Adaptive (BESS - parent) | Adaptive (BESS - provider) |
|  |  |  |  |  |  |  |
| Q2 | -0.28* | -0.09 | -0.19 | -0.16 | 0.35** | 0.10 |
|  | (-0.54 - -0.03) | (-0.41 - 0.23) | (-0.46 - 0.07) | (-0.47 - 0.16) | (0.11 - 0.58) | (-0.21 - 0.41) |
| Q3 | -0.31* | -0.37* | -0.17 | -0.31 | 0.42** | 0.30 |
|  | (-0.58 - -0.03) | (-0.71 - -0.02) | (-0.46 - 0.11) | (-0.64 - 0.03) | (0.17 - 0.68) | (-0.03 - 0.63) |
| Q4 | -0.29* | -0.27 | -0.15 | -0.14 | 0.51*** | 0.19 |
|  | (-0.58 - -0.00) | (-0.63 - 0.08) | (-0.45 - 0.14) | (-0.49 - 0.21) | (0.24 - 0.77) | (-0.15 - 0.53) |
|  |  |  |  |  |  |  |
| N | 574 | 411 | 574 | 411 | 574 | 411 |
| R-sq. | 0.06 | 0.13 | 0.03 | 0.05 | 0.10 | 0.18 |

Note. 95% confidence intervals in parentheses. All models include as covariates age, age-sq, gender, race/ethnicity, respondent’s spouse lives at home, total household members, provider type

*** *p*<.001, ** *p*<.01, * *p*<.05
