# Supplementary material for: Executive function, self-regulation skills, behaviors, and socioeconomic status in early childhood
Source: PLoS One. 2022 Nov 2;17(11):e0277013. doi: 10.1371/journal.pone.0277013 (PMC9629624; doi:10.1371/journal.pone.0277013)
Supplement: S8 Table — (DOCX) [file pone.0277013.s008.docx]

S8 Table. Average SES effects in executive function for children aged 43-50 months

|  | (1) | (2) |
| --- | --- | --- |
| VARIABLES | EF (MEFS) | Inhibitory control (PT) |
|  |  |  |
| Q2 | 0.09 | 0.12 |
|  | (-0.12 - 0.30) | (-0.10 - 0.34) |
| Q3 | 0.26* | 0.18 |
|  | (0.02 - 0.49) | (-0.06 - 0.42) |
| Q4 | 0.48*** | 0.14 |
|  | (0.24 - 0.72) | (-0.11 - 0.39) |
|  |  |  |
| N | 642 | 667 |
| R-sq. | 0.11 | 0.12 |

Note. 95% confidence intervals in parentheses. All models include as covariates age, age-sq, gender, race/ethnicity, respondent’s spouse lives at home, total household members, provider type

*** *p*<.001, ** *p*<.01, * *p*<.05
