# Supplementary material for: Executive function, self-regulation skills, behaviors, and socioeconomic status in early childhood
Source: PLoS One. 2022 Nov 2;17(11):e0277013. doi: 10.1371/journal.pone.0277013 (PMC9629624; doi:10.1371/journal.pone.0277013)
Supplement: S9 Table — (DOCX) [file pone.0277013.s009.docx]

S9 Table. Average SES effects in self-regulation skills for children aged 43-50 months

|  | (1) | (2) | (3) | (4) |
| --- | --- | --- | --- | --- |
| VARIABLES | Regulation (Leiter-Cog/Soc) | Regulation (Leiter Emo/Reg) | Dysregulation (BRIEF - parent) | Dysregulation (BRIEF - provider) |
|  |  |  |  |  |
| Q2 | 0.04 | 0.00 | -0.04 | -0.32* |
|  | (-0.17 - 0.25) | (-0.21 - 0.22) | (-0.26 - 0.18) | (-0.59 - -0.06) |
| Q3 | 0.01 | -0.03 | -0.03 | -0.35* |
|  | (-0.23 - 0.25) | (-0.28 - 0.21) | (-0.29 - 0.22) | (-0.66 - -0.04) |
| Q4 | 0.24 | 0.12 | -0.23 | -0.39* |
|  | (-0.01 - 0.49) | (-0.13 - 0.37) | (-0.49 - 0.03) | (-0.71 - -0.08) |
|  |  |  |  |  |
| N | 756 | 756 | 749 | 519 |
| R-sq. | 0.09 | 0.05 | 0.05 | 0.07 |

Note. 95% confidence intervals in parentheses. All models include as covariates age, age-sq, gender, race/ethnicity, respondent’s spouse lives at home, total household members, provider type

*** *p*<.001, ** *p*<.01, * *p*<.05
