# Supplementary material for: Executive function, self-regulation skills, behaviors, and socioeconomic status in early childhood
Source: PLoS One. 2022 Nov 2;17(11):e0277013. doi: 10.1371/journal.pone.0277013 (PMC9629624; doi:10.1371/journal.pone.0277013)
Supplement: S10 Table — (DOCX) [file pone.0277013.s010.docx]

S10 Table. Average SES effects in behaviors for children aged 43-50 months

|  | (1) | (2) | (3) | (4) | (5) | (6) |
| --- | --- | --- | --- | --- | --- | --- |
| VARIABLES | Externalizing (BESS - parent) | Externalizing (BESS -provider) | Internalizing (BESS - parent) | Internalizing (BESS - provider) | Adaptive (BESS - parent) | Adaptive (BESS - provider) |
|  |  |  |  |  |  |  |
| Q2 | -0.22 | -0.28* | -0.05 | -0.15 | 0.34** | 0.34* |
|  | (-0.44 - 0.01) | (-0.54 - -0.02) | (-0.27 - 0.17) | (-0.42 - 0.13) | (0.12 - 0.56) | (0.08 - 0.60) |
| Q3 | -0.17 | -0.22 | 0.05 | -0.17 | 0.38** | 0.48** |
|  | (-0.42 - 0.09) | (-0.51 - 0.08) | (-0.21 - 0.31) | (-0.49 - 0.15) | (0.12 - 0.63) | (0.18 - 0.78) |
| Q4 | -0.41** | -0.27 | -0.14 | 0.03 | 0.46*** | 0.59*** |
|  | (-0.68 - -0.15) | (-0.58 - 0.03) | (-0.41 - 0.12) | (-0.29 - 0.36) | (0.21 - 0.72) | (0.29 - 0.90) |
|  |  |  |  |  |  |  |
| N | 754 | 520 | 754 | 520 | 756 | 520 |
| R-sq. | 0.05 | 0.07 | 0.03 | 0.04 | 0.07 | 0.11 |

Note. 95% confidence intervals in parentheses. All models include as covariates age, age-sq, gender, race/ethnicity, respondent’s spouse lives at home, total household members, provider type

*** *p*<.001, ** *p*<.01, * *p*<.05
