# Supplementary material for: Executive function, self-regulation skills, behaviors, and socioeconomic status in early childhood
Source: PLoS One. 2022 Nov 2;17(11):e0277013. doi: 10.1371/journal.pone.0277013 (PMC9629624; doi:10.1371/journal.pone.0277013)
Supplement: S11 Table — (DOCX) [file pone.0277013.s011.docx]

S11 Table. Average SES effects in executive function for children aged 51-60 months

|  | (1) | (2) |
| --- | --- | --- |
| VARIABLES | EF (MEFS) | Inhibitory control (PT) |
|  |  |  |
| Q2 | 0.27* | 0.06 |
|  | (0.04 - 0.51) | (-0.13 - 0.25) |
| Q3 | 0.62*** | 0.30** |
|  | (0.36 - 0.88) | (0.10 - 0.51) |
| Q4 | 0.46** | 0.31** |
|  | (0.18 - 0.75) | (0.08 - 0.54) |
|  |  |  |
| N | 548 | 568 |
| R-sq. | 0.16 | 0.13 |

Note. 95% confidence intervals in parentheses. All models include as covariates age, age-sq, gender, race/ethnicity, respondent’s spouse lives at home, total household members, provider type

*** *p*<.001, ** *p*<.01, * *p*<.05
