# Supplementary material for: Executive function, self-regulation skills, behaviors, and socioeconomic status in early childhood
Source: PLoS One. 2022 Nov 2;17(11):e0277013. doi: 10.1371/journal.pone.0277013 (PMC9629624; doi:10.1371/journal.pone.0277013)
Supplement: S12 Table — (DOCX) [file pone.0277013.s012.docx]

S12 Table. Average SES effects in self-regulation skills for children aged 51-60 months

|  | (1) | (2) | (3) | (4) |
| --- | --- | --- | --- | --- |
| VARIABLES | Regulation (Leiter-Cog/Soc) | Regulation (Leiter Emo/Reg) | Dysregulation (BRIEF - parent) | Dysregulation (BRIEF - provider) |
|  |  |  |  |  |
| Q2 | 0.19* | 0.18* | -0.22 | -0.19 |
|  | (0.01 - 0.36) | (0.00 - 0.36) | (-0.44 - 0.01) | (-0.44 - 0.07) |
| Q3 | 0.26** | 0.19 | -0.14 | -0.25 |
|  | (0.07 - 0.46) | (-0.01 - 0.39) | (-0.39 - 0.11) | (-0.54 - 0.03) |
| Q4 | 0.34** | 0.22 | -0.27 | -0.57*** |
|  | (0.13 - 0.56) | (-0.00 - 0.44) | (-0.55 - 0.01) | (-0.89 - -0.24) |
|  |  |  |  |  |
| N | 645 | 645 | 651 | 432 |
| R-sq. | 0.07 | 0.04 | 0.09 | 0.15 |

Note. 95% confidence intervals in parentheses. All models include as covariates age, age-sq, gender, race/ethnicity, respondent’s spouse lives at home, total household members, provider type

*** *p*<.001, ** *p*<.01, * *p*<.05
