# Supplementary material for: Executive function, self-regulation skills, behaviors, and socioeconomic status in early childhood
Source: PLoS One. 2022 Nov 2;17(11):e0277013. doi: 10.1371/journal.pone.0277013 (PMC9629624; doi:10.1371/journal.pone.0277013)
Supplement: S13 Table — (DOCX) [file pone.0277013.s013.docx]

S13 Table. Average SES effects in behaviors for children aged 51-60 months

|  | (1) | (2) | (3) | (4) | (5) | (6) |
| --- | --- | --- | --- | --- | --- | --- |
| VARIABLES | Externalizing (BESS - parent) | Externalizing (BESS -provider) | Internalizing (BESS - parent) | Internalizing (BESS - provider) | Adaptive (BESS - parent) | Adaptive (BESS - provider) |
|  |  |  |  |  |  |  |
| Q2 | -0.37** | -0.29* | -0.12 | -0.17 | 0.55*** | 0.15 |
|  | (-0.60 - -0.15) | (-0.56 - -0.01) | (-0.35 - 0.12) | (-0.47 - 0.13) | (0.32 - 0.78) | (-0.11 - 0.40) |
| Q3 | -0.30* | -0.27 | 0.04 | -0.13 | 0.60*** | 0.25 |
|  | (-0.55 - -0.05) | (-0.58 - 0.03) | (-0.23 - 0.30) | (-0.46 - 0.21) | (0.34 - 0.85) | (-0.04 - 0.53) |
| Q4 | -0.23 | -0.43* | 0.04 | -0.42* | 0.71*** | 0.61*** |
|  | (-0.51 - 0.05) | (-0.78 - -0.08) | (-0.26 - 0.33) | (-0.80 - -0.04) | (0.43 - 1.00) | (0.28 - 0.93) |
|  |  |  |  |  |  |  |
| N | 652 | 432 | 652 | 432 | 652 | 432 |
| R-sq. | 0.08 | 0.13 | 0.03 | 0.08 | 0.12 | 0.14 |

Note. 95% confidence intervals in parentheses. All models include as covariates age, age-sq, gender, race/ethnicity, respondent’s spouse lives at home, total household members, provider type

*** *p*<.001, ** *p*<.01, * *p*<.05
